# Supplementary material for: Energetics and evasion dynamics of large predators and prey: pumas vs. hounds
Source: PeerJ. 2017 Aug 17;5:e3701. doi: 10.7717/peerj.3701 (PMC5563439; doi:10.7717/peerj.3701)
Supplement: Table S1 [file peerj-05-3701-s002.docx]

| **Table S1:** Total number of observed evasive puma behaviors (pooled across all 4 escapes). | | | | |
| --- | --- | --- | --- | --- |
| Evasive puma behavior |  | Definition |  | No. observations |
| Treeing |  | Jumping into tree |  | 5 |
| Hairpin turn |  | Execution of turn >135 degrees |  | 4 |
| Figure-of-8 maneuver |  | Complete “8” pattern run on landscape |  | 1 |
| Fleeing uphill |  | Running >10m up an incline > 30 degree slope |  | 4 |
