# Supplementary figures and images for: Energetics and evasion dynamics of large predators and prey: pumas vs. hounds
Source: PeerJ. 2017 Aug 17;5:e3701. doi: 10.7717/peerj.3701 (PMC5563439; doi:10.7717/peerj.3701)

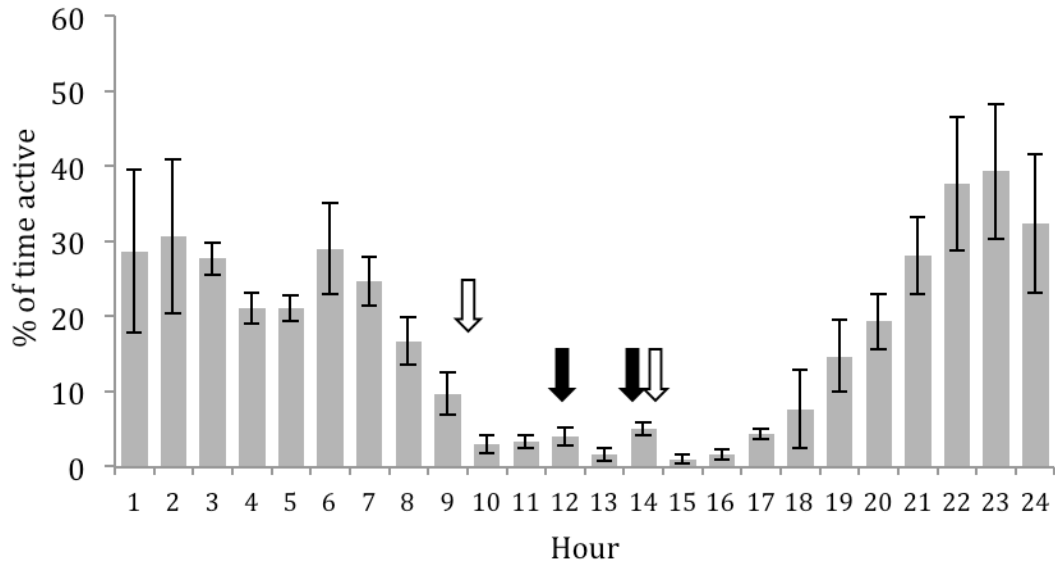

Supplement: Figure S1 — Arrows correspond to the time of day in which chases for recapture occurred. White arrows show the timing of chases for male puma 36 on Aug. 3, 2015, and black arrows show the timing of chases for male puma 26 on Nov. 18, 2015. Daily activity estimates are averaged across 2 weeks from each of three adult male pumas (Wang, Allen & Wilmers, 2015). [file peerj-05-3701-s003.pdf]

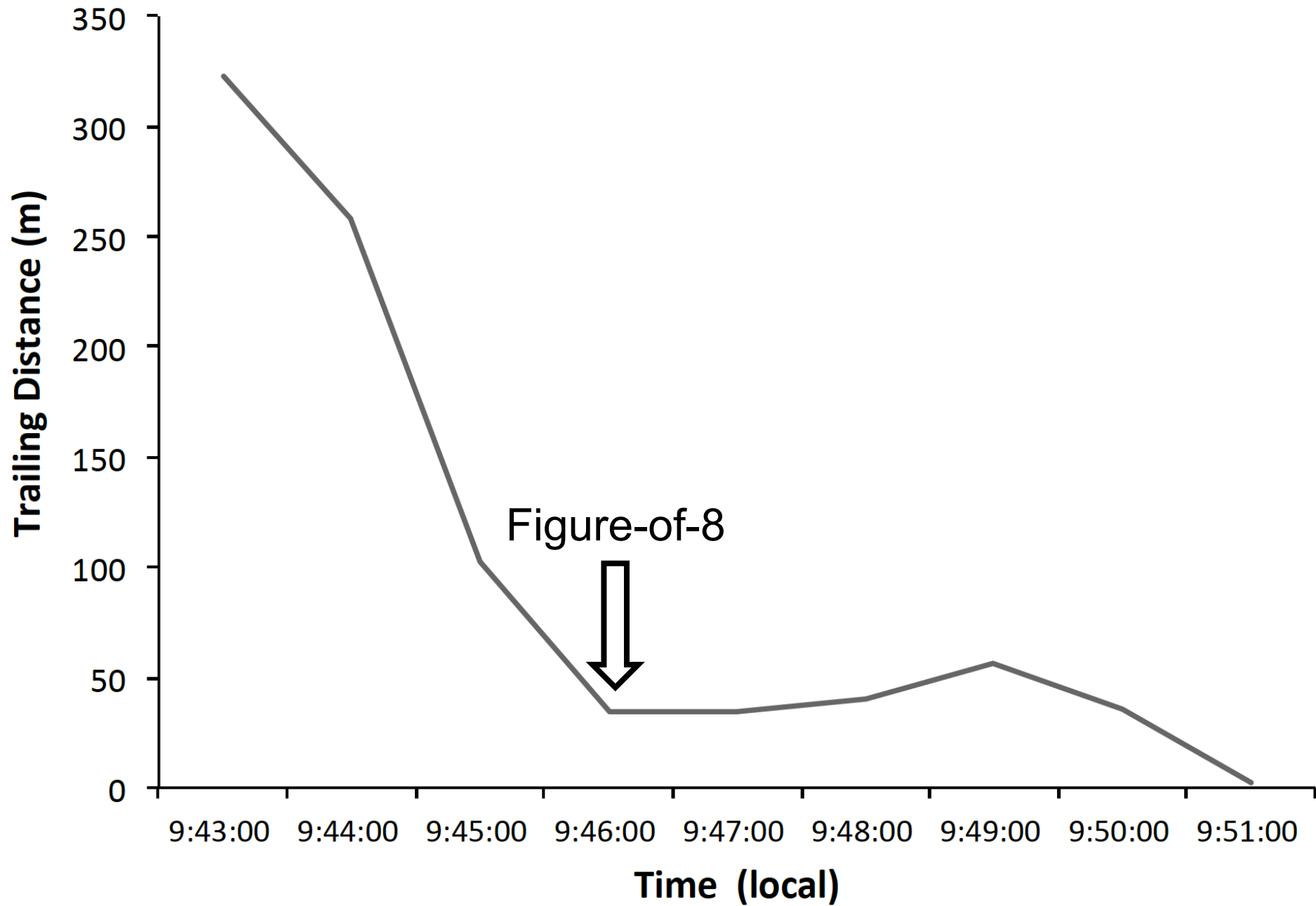

Supplement: Figure S5 — Trailing distance (m) of hounds rapidly decreased until 9:46:00, when 26 M ran a figure-of-8 pattern and briefly escaped into a tree. This maneuver increased separation distance by nearly 15 m, and the puma’s capture was delayed by an additional 5 min. [file peerj-05-3701-s007.pdf]
