# Supplementary material for: Energetics and evasion dynamics of large predators and prey: pumas vs. hounds
Source: PeerJ. 2017 Aug 17;5:e3701. doi: 10.7717/peerj.3701 (PMC5563439; doi:10.7717/peerj.3701)
Supplement: Figure S2 — Insets display ODBA (g, B), speed (ms−1, C), and estimated mass-specific metabolic demand ($\dot {\mathrm{V }}{\mathrm{O}}_{2}$V ˙O2 in ml O2kg−1min−1, D.) For B, C, and D, mean values are presented as dashed horizontal lines, and solid horizontal lines in D. depict $\dot {\mathrm{V }}{\mathrm{O}}_{2\mathrm{MAX}}$V ˙O2MAX for each species. The elevation profile (F) for the accelerometer-GPS-equipped hound is also presented. Map data©2016 Google. [file peerj-05-3701-s004.pdf]

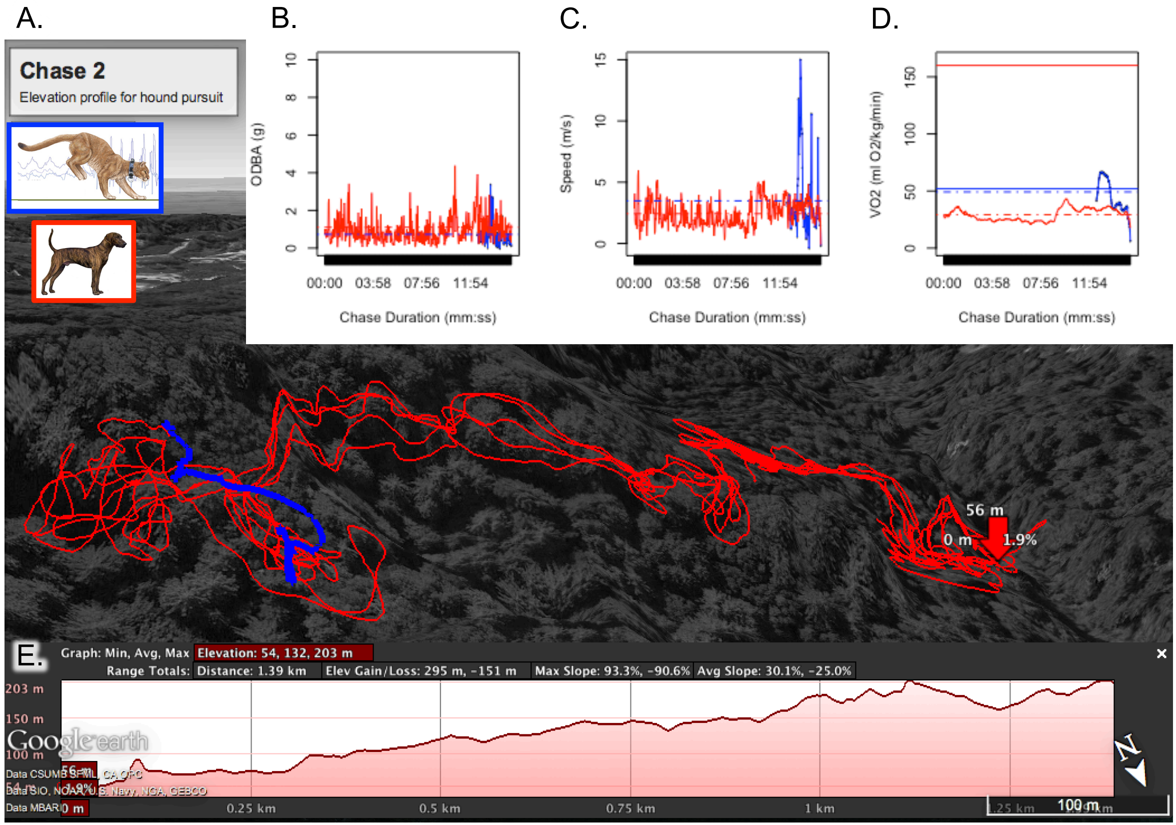

A.
B.
C.
D.
Chase 2
Elevation profile for hound pursuit
ODBA (g)
Speed (m/s)
VO2 (ml O2/kg/min)
Chase Duration (mm:ss)
00:00
03:58
07:56
11:54
56 m
0 m
1.9%
E.
Graph: Min, Avg, Max
Elevation: 54, 132, 203 m
Range Totals:
Distance: 1.39 km
Elev Gain/Loss: 295 m, -151 m
Max Slope: 93.3%, -90.6%
Avg Slope: 30.1%, -25.0%
203 m
150 m
100 m
0.25 km
0.5 km
0.75 km
1 km
100 m
Google earth
Data CSUMB SFML, CA OPC
Data SIO, NOAA, U.S. Navy, NGA, GEBCO
Data MBARI
