# Supplementary material for: Energetics and evasion dynamics of large predators and prey: pumas vs. hounds
Source: PeerJ. 2017 Aug 17;5:e3701. doi: 10.7717/peerj.3701 (PMC5563439; doi:10.7717/peerj.3701)
Supplement: Figure S3 — Insets display ODBA (g, B), speed (ms−1, C), and estimated mass-specific metabolic demand ($\dot {\mathrm{V }}{\mathrm{O}}_{2}$V ˙O2 in ml O2kg−1min−1, D) For B, C, and D, mean values are presented as dashed horizontal lines, and solid horizontal lines in D depict $\dot {\mathrm{V }}{\mathrm{O}}_{2\mathrm{MAX}}$V ˙O2MAX for each species. The elevation profile (F) for the accelerometer-GPS-equipped hound is also presented. Map data©2016 Google. [file peerj-05-3701-s005.pdf]

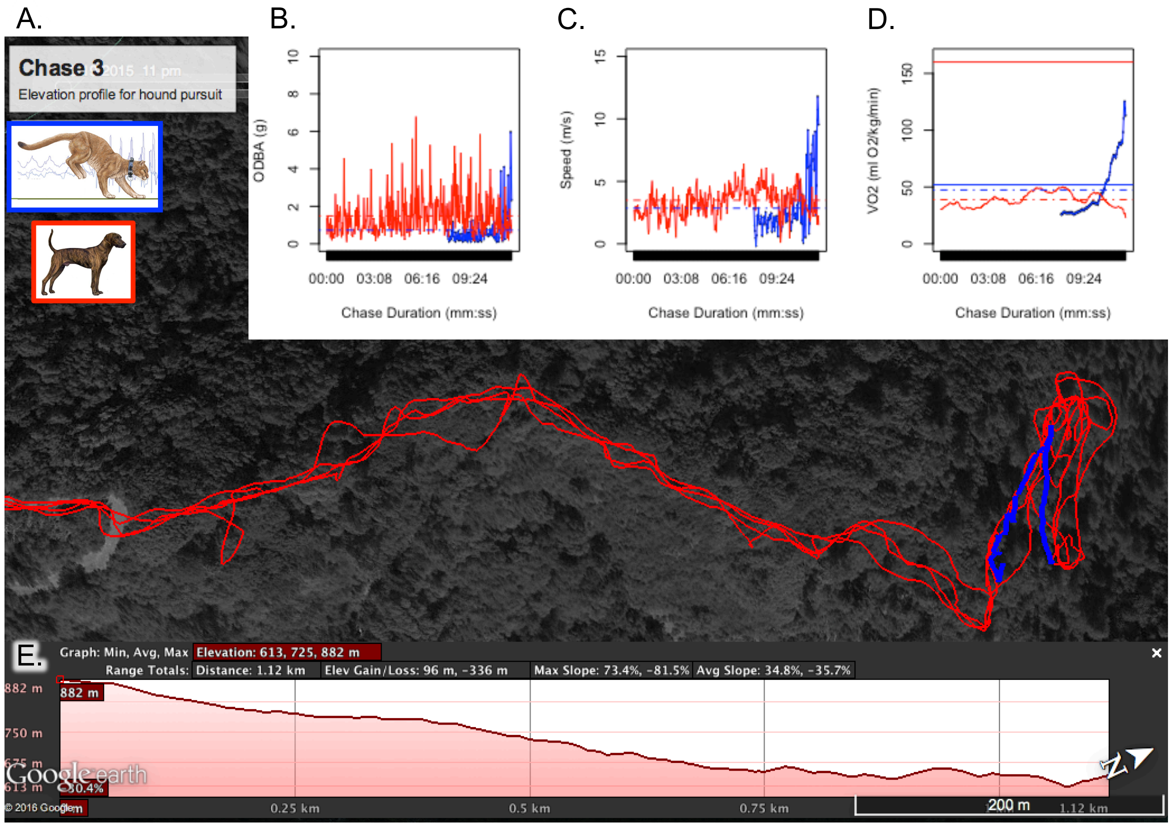
A.
Chase 3
Elevation profile for hound pursuit
B.
ODBA (g)
Chase Duration (mm:ss)
C.
Speed (m/s)
Chase Duration (mm:ss)
D.
VO2 (ml O2/kg/min)
Chase Duration (mm:ss)
00:00 03:08 06:16 09:24
E.
Graph: Min, Avg, Max
Elevation: 613, 725, 882 m
Range Totals:
Distance: 1.12 km
Elev Gain/Loss: 96 m, -336 m
Max Slope: 73.4%, -81.5%
Avg Slope: 34.8%, -35.7%
882 m
750 m
675 m
613 m
0.25 km
0.5 km
0.75 km
1.12 km
200 m
Google earth
© 2016 Google
