# Supplementary material for: Energetics and evasion dynamics of large predators and prey: pumas vs. hounds
Source: PeerJ. 2017 Aug 17;5:e3701. doi: 10.7717/peerj.3701 (PMC5563439; doi:10.7717/peerj.3701)
Supplement: Figure S4 — Insets display ODBA (g, B), speed (ms−1, C), and estimated mass-specific metabolic demand ($\dot {\mathrm{V }}{\mathrm{O}}_{2}$V ˙O2 in ml O2kg−1min−1, D) For B, C, and D, mean values are presented as dashed horizontal lines, and solid horizontal lines in D. depict $\dot {\mathrm{V }}{\mathrm{O}}_{2\mathrm{MAX}}$V ˙O2MAX for each species. The elevation profile (F) for the accelerometer-GPS-equipped hound is also presented. Map data©2016 Google. [file peerj-05-3701-s006.pdf]

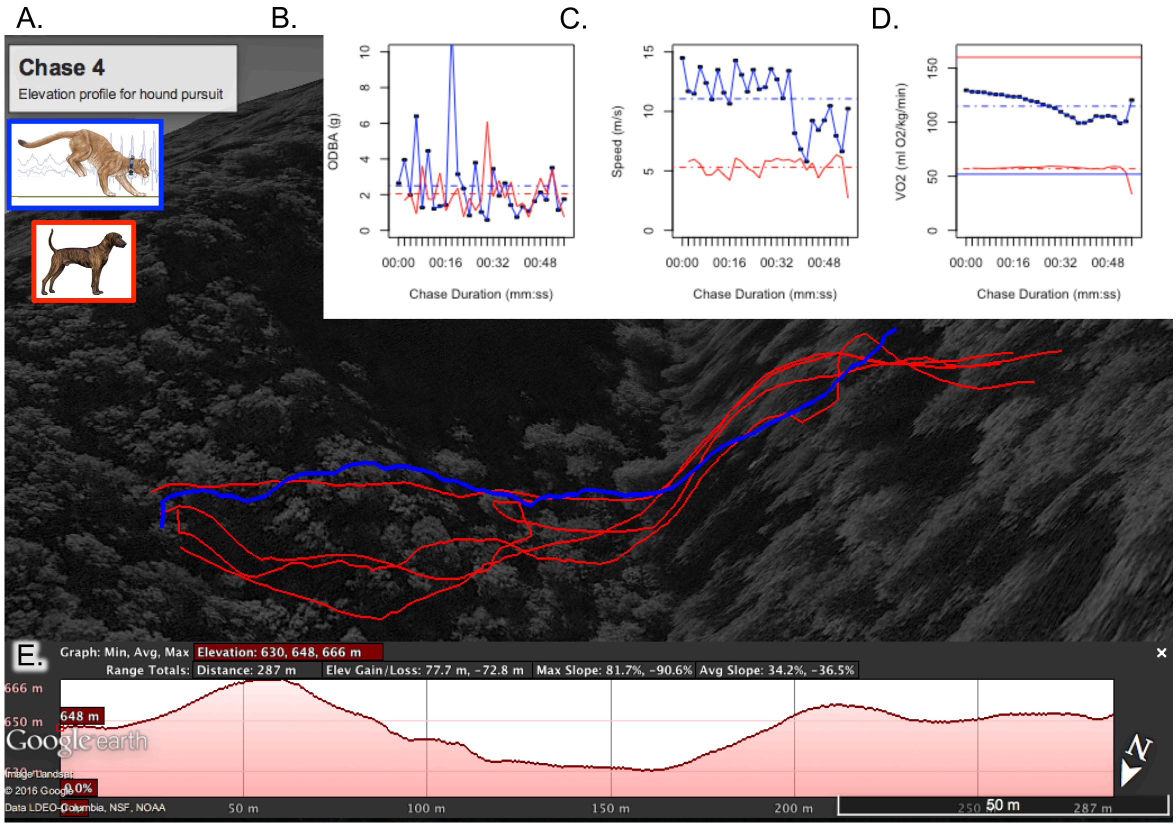
A.
B.
C.
D.
Chase 4
Elevation profile for hound pursuit
ODBA (g)
Speed (m/s)
VO2 (ml O2/kg/min)
Chase Duration (mm:ss)
00:00 00:16 00:32 00:48
E.
Graph: Min, Avg, Max
Elevation: 630, 648, 666 m
Range Totals:
Distance: 287 m
Elev Gain/Loss: 77.7 m, -72.8 m
Max Slope: 81.7%, -90.6%
Avg Slope: 34.2%, -36.5%
666 m
650 m
648 m
50 m
100 m
150 m
200 m
250 m
287 m
Google earth
Image Landsat
© 2016 Google
Data LDEO-Columbia, NSF, NOAA
